# Association of statewide stay-at-home orders with utilization of case management and supportive services for veterans experiencing housing insecurity

**DOI:** 10.1038/s44184-022-00010-x

**Published:** 2022-08-26

**Authors:** Eric Jutkowitz, Christopher Halladay, Jack Tsai, Dina Hooshyar, Portia Y. Cornell, James L. Rudolph

**Affiliations:** 1 Center of Innovation in Long Term Services and Supports, Providence VA Medical Center, Providence, RI USA; 2 Department of Health Services, Policy & Practice, Brown University School of Public Health, Providence, RI USA; 3 Evidence Synthesis Program Center, Providence VA Health Care System, Providence, RI USA; 4 VA National Center on Homelessness among Veterans, Tampa, FL USA; 5 School of Public Health, University of Texas Health Science Center at Houston, Houston, TX USA; 6 Department of Psychiatry, University of Texas Southwestern Medical Center, Dallas, TX USA

**Keywords:** Risk factors, Medical research, Outcomes research

## Abstract

The US Department of Housing and Urban Development-Department of Veterans Affairs (VA) Supportive Housing (HUD-VASH) program provides Veterans with a subsidy for rent and case management. In response to the Coronavirus 2019 pandemic, many states enacted stay-at-home orders that may have limited access to case managers. Therefore, we examined the association between statewide stay-at-home orders and utilization of HUD-VASH case management. We linked data on whether a state implemented a statewide stay-at-home order between March 1, 2020 and April 30, 2020 with VA medical records. Analysis time was centered on the date of a state’s stay-at-home order (exposed states). For Veterans in states without a stay-at home-order (unexposed states), we used the average date exposed states implemented an order (March 27, 2020). We used a difference-in-difference design and adjusted linear regression models to compare total, in-person, telephone, and video case management encounters per Veteran in the 60 days after a stay-at-home order relative to the prior year. There was no significant difference in utilization of case management between Veterans who lived in states that did and did not issue a stay-at-home order. Across all states and in the 60 days after the index date relative to the prior year, Veterans had more total, telephone and video, and fewer in-person encounters. Statewide stay-at-home orders did not differentially affect utilization of case management. Virtual case management in HUD-VASH can increase program reach; however, the effect of virtual case management on outcomes such as quality of life and Veteran satisfaction is unknown.

## Introduction

The Coronavirus 2019 (COVID-19) pandemic has caused 24% of American households to experience housing insecurity (rent or mortgage nonpayment or uncertainty about ability to make payments), and housing insecurity is associated with decreased access to healthcare^1,2^.

Veterans are one of the largest populations in the United States who are at risk of experiencing housing insecurity^3^. The US Department of Housing and Urban Development-VA Supportive Housing (HUD-VASH) is one program to end Veteran homelessness. HUD-VASH provided nearly 80,000 Veterans with a subsidy for rent at the end of 2020^4^. Veterans in HUD-VASH are responsible for only 30% of their rent^5^. As part of the HUD-VASH program, Department of Veterans Affairs (VA) staff provide Veterans with at least monthly or bi-monthly case management contacts that teach them life skills, link them with VA and community services and stabilize their mental health^4^. HUD-VASH case management is associated with increased access to VA healthcare services and increased use of primary care^6,7^. HUD-VASH is the largest supported housing program for Veterans in the country, and its expansion over the past 10 years has contributed to a major reduction in Veteran homelessness^8^.

In response to the COVID-19 pandemic, many states enacted stay-at-home orders, which may have limited access to transportation, healthcare services and HUD-VASH case managers^9^. Concurrently, many VA medical centers transitioned HUD-VASH case management to virtual healthcare (telephone and video), but this method of care delivery may be inaccessible to Veterans experiencing housing insecurity^10^. The VA implemented several steps to help the most economically insecure Veterans continue to receive care during the transition to virtual healthcare^11^. First, Veteran facing staff received telehealth training. Second, HUD-VASH case managers increased outreach efforts and proactively contacted Veterans. Simultaneously, all frontline clinicians were tasked with identifying and contacting high risk populations in primary care and mental health. Third, the Homeless Program Office used CARES funding to distribute over 20,000 free smartphones to Veterans experiencing housing insecurity. Fourth, the VA Office of Rural Health funded various initiatives to help rural Veterans access virtual mental health services. Prior to the COVID-19 pandemic, HUD-VASH clinical teams typically delivered care in the community, at Veterans’ homes, or at VA medical centers. To inform HUD-VASH care delivery, it is important to understand how stay-at-home orders and change in care management delivery from in-person community care to virtual care may have affected the utilization of case management.

We evaluated the association between statewide stay-at-home orders and the frequency and modality (in-person, telehealth, and video-health) of HUD-VASH case management encounters. Following the VA’s COVID-19 Response Plan that encouraged medical centers to maintain care through telehealth^10^, we believe that HUD-VASH sites and case managers implemented virtual methods of contact independent of state stay-at-home policies. We expect that, on average, Veterans who lived in states that implemented a stay-at-home order did not differentially use case management services than Veterans who lived in states that did not implement a stay-at-home order.

## Methods

### Data and Sample

We used public data to determine which states and the District of Columbia implemented a statewide stay-at-home (includes shelter-in-place) order between March 1, 2020 and April 30, 2020^9^. We linked these data with data from the VA’s Corporate Data Warehouse and Homeless Operations Management and Evaluation System, which provide information on Veteran enrollment, demographics, and medical records. We assigned Veterans to a state of residence based on the location of the VA Medical Center that delivered their HUD-VASH case management.

Analysis time was indexed on the date of a state’s stay-at-home order (exposed; *n* = 43). Unexposed states (*n* = 8) did not implement a stay-at-home order, so we assigned Veterans in these states an index date of March 27, 2020, which is the average date exposed states implemented an order.

We included Veterans who were enrolled in HUD-VASH at any time in 60 days after the index date or the prior year (−360 to −300 before the index date). We censored observations from Veterans during the months they were not enrolled in HUD-VASH and excluded Veterans with multiple enrollment dates without a separating disenrollment and who had missing data on covariates described below.

We follow the STROBE Statement for observational studies study and our study was approved by the Providence VA Medical Center Institutional Review Board and Research and Development committees.

### Outcomes

We used the VA Managerial Cost Accounting Stop Codes to identify the total number of HUD-VASH encounters per Veteran in 60-day intervals before and after the index date. We also used Stop Codes to determine whether each case management encounter was in-person or via telephone or video. Finally, we calculated the proportion of Veterans with no HUD-VASH encounter within each 60-day period before and after the index date.

### Covariates

Veteran sociodemographic and clinical characteristics were obtained from the VA Corporate Data Warehouse. Specifically, we obtained each Veteran’s age, sex, race, whether they served in combat, whether they had a service-connected disability, and marital status on the month of enrollment in HUD-VASH. We used the Elixhauser algorithm to determine whether a Veteran had a diagnosis (yes/no) of rheumatic disease, renal disease, liver disease, depression, diabetes, hypertension, congestive heart failure, valvular disease, pulmonary disease, and HIV^12^. We used VA Informatics and Computing Infrastructure definitions to determine whether a Veteran had a diagnosis of substance use disorder, post-traumatic stress disorder, and traumatic brain injury and the Chronic Conditions Warehouse definition to determine whether a Veteran had Alzheimer’s disease^13^.

### Analysis

Analyses were conducted at the Veteran level. We graphed unadjusted average total, in-person, telephone and video HUD-VASH encounters, and the proportion of Veterans with no HUD-VASH encounter within each 60-day period before and after the index date.

We made all statistical comparisons of utilization between the 60 days after the index date relative to the same period in the prior year. We used a difference-in-differences design and adjusted linear regression models to compare encounters (separate models per outcome) among Veterans in 43 states with a stay-at-home order to Veterans in 8 states without a stay-at-home order. From the adjusted linear regressions, we also calculated the change in number of encounters per Veteran. We controlled for Veteran demographics (age, sex, race, whether the Veteran served in combat, service-connected disability, and marital status) and individual chronic conditions which we believe may benefit from HUD-VASH case management or the ability to engage in virtual care. We obtained standard errors accounting for clustering within states.

## Results

### Sample characteristics

We observed 56,682 Veterans meeting the inclusion criteria. The mean (standard deviation) age of Veterans was 53.1 (12.8) years; 13% were women, 51% were white, 39% were black, 0.7% were Asian, and 9% were other races (American Indian, Alaskan Native, Asian, Native Hawaiian, other Pacific Islander, or missing/unknown; Table 1). Veterans who lived in states that implemented a stay-at-home order compared to those who lived in states that did not implement an order were more racially diverse (*p* < 0.001), less likely to live in a rural area (*n* = 6,755 [12.6%] vs. *n* = 613 [19.2%]; *p* < 0.001), more likely to have a service connected disability (*n* = 6,952 [13%] vs. *n* = 329 [10.3%]; *p* < 0.001), less likely to have alcohol (*n* = 20,063 [37.5%] vs. *n* = 1361 (42.6%); *p* < 0.001) or drug (*n* = 20,670 [38.6%] vs. *n* = 1,325 [41.5%]; *p* = 0.001) diagnoses and less likely to be diagnosed with depression (*n* = 27,127 [50.7%] vs. *n* = 1,810 [56.7%]; *p* < 0.001).

**Table 1:** Characteristics of Veterans in states with and without a stay-at-home order^a^.

	Overall	Stay-at-home	No stay-at-home	*P*-value stay-at-home vs. no stay-at-home
States and District of Columbia	51	43	8	
Unique number of Veterans	56,682	53,487	3195	
Age, mean (SD)	53.1 (12.8)	53.2 (12.8)	52.7 (11.9)	0.077
Female, *n* (%)	7231 (12.8%)	6857 (12.8%)	374 (11.7%)	0.067
Race, *n* (%)^a^				<0.001
White	29302 (51.7%)	27089 (50.6%)	2213 (69.3%)	
African American	21841 (38.5%)	21228 (39.7%)	613 (19.2%)	
Asian	384 (0.7%)	373 (0.7%)	suppressed^b^	
Other	5155 (9.1%)	4797 (9.0%)	suppressed^b^	
Rural, *n* (%)	7368 (13.0%)	6755 (12.6%)	613 (19.2%)	<0.001
Served in combat, *n* (%)	9311 (16.4%)	8819 (16.5%)	492 (15.4%)	0.11
Service-connected disability, *n* (%)	7281 (12.8%)	6952 (13.0%)	329 (10.3%)	<0.001
Married, *n* (%)	7095 (12.5%)	6741 (12.6%)	354 (11.1%)	0.011
Alcohol use disorder	21424 (37.8%)	20063 (37.5%)	1361 (42.6%)	<0.001
Drug use disorder	21995 (38.8%)	20670 (38.6%)	1325 (41.5%)	0.001
Rheumatic disease	1044 (1.8%)	986 (1.8%)	58 (1.8%)	0.91
Renal disease	3269 (5.8%)	3102 (5.8%)	167 (5.2%)	0.18
Liver disease	7418 (13.1%)	7016 (13.1%)	402 (12.6%)	0.38
Depression	28,937 (51.1%)	27127 (50.7%)	1810 (56.7%)	<0.001
Diabetes	11204 (19.8%)	10546 (19.7%)	658 (20.6%)	0.23
Hypertension	25802 (45.5%)	24277 (45.4%)	1525 (47.7%)	0.010
Congestive heart failure	3552 (6.3%)	3366 (6.3%)	186 (5.8%)	0.29
Valvular disease	1270 (2.2%)	1194 (2.2%)	76 (2.4%)	0.59
Pulmonary disease	11314 (20.0%)	10586 (19.8%)	728 (22.8%)	<0.001
HIV	1004 (1.8%)	978 (1.8%)	26 (0.8%)	<0.001
Post-traumatic stress disorder	17602 (31.1%)	16586 (31.0%)	1016 (31.8%)	0.35
Psychoses	15961 (28.2%)	15041 (28.1%)	920 (28.8%)	0.41
Traumatic brain injury	2760 (4.9%)	2597 (4.9%)	163 (5.1%)	0.53
Alzheimer’s disease and related dementias	1060 (1.9%)	992 (1.9%)	68 (2.1%)	0.27

^a^Other race includes: American Indian or Alaskan Native, Asian, Native Hawaiian or other Pacific Islander, or missing/unknown.

^b^We suppressed the cell due to small sample size.

### Case management encounters

Trends in the unadjusted number of encounters and the proportion of Veterans with no HUD-VASH encounter before and after the index date were similar among states that did and did not issue an order (Supplementary Figs 1–5). Across all states and in the 60 days after the index date relative to the prior year, Veterans had more HUD-VASH encounters (Table 2): total (adjusted change 1.43; 95%CI: 1.10, 1.75), telephone (adjusted change: 2.54; 95%CI: 2.24, 2.85) and video (adjusted change: 0.07; 95%CI: 0.04, 0.10) and fewer in-person encounters (adjusted change: −1.17; 95%CI: −1.44, −0.91). The proportion of Veterans with no HUD-VASH encounter decreased by 13 percentage points (95%CI: −19, −9) in the 60 days after the index date compared to the prior year.

**Table 2:** Encounters per Veterans in 60 days after and −360 to −300 days before index date (*n* = 56,682).

Outcome	Mean (SD) −360 to −300 days before the index date	Mean (SD) 0 to 60 days post index date	Adjusted pre-post change (95% CI)	Adjusted difference-in-differences (95% CI)
*Number of Total Encounters*				
Overall	3.02 (3.51)	4.46 (4.24)	1.43 (1.10, 1.75)	
Stay-at-home order	3.02 (3.54)	4.48 (4.26)	1.43 (1.10, 1.76)	0.29 (−0.60, 1.18)
No stay-at-home order	2.93 (3.11)	4.10 (3.93)	1.14 (0.31, 1.97)	
*Number of In-Person Encounters*				
Overall	2.15 (2.71)	0.97 (2.03)	−1.17 (−1.44, −0.91)	
Stay-at-home order	2.15 (2.72)	0.97 (2.05)	−1.19 (−1.46, −0.91)	0.01 (−0.65, 0.67)
No stay-at-home order	2.16 (2.38)	0.98 (1.68)	−1.19 (−1.81, −0.58)	
*Number of Telephone Encounters*				
Overall	0.87 (1.68)	3.41 (3.59)	2.54 (2.24, 2.85)	
Stay-at-home order	0.88 (1.69)	3.43 (3.61)	2.55 (2.22, 2.87)	0.26 (−0.32, 0.83)
No stay-at-home order	0.77 (1.51)	3.08 (3.22)	2.29 (1.81, 2.77)	
*Number of Video Encounters*				
Overall	0.00 (0.06)	0.07 (0.49)	0.07 (0.04, 0.10)	
Stay-at-home order	0.00 (0.05)	0.07 (0.49)	0.07 (0.04, 0.10)	0.02 (−0.02, 0.07)
No stay-at-home order	0.01 (0.09)	0.05 (0.33)	0.05 (0.02, 0.80)	
*Proportion of Veterans with No Encounter*				
Overall	0.24 (0.43)	0.10 (0.31)	−0.13 (−0.19, −0.09)	
Stay-at-home order	0.24 (0.43)	0.11 (0.31)	−0.13 (−0.18, −0.09)	−0.035 (−0.13, 0.06)
No stay-at-home order	0.19 (0.39)	0.09 (0.29)	−0.10 (−0.18, −0.02)	

*SD* standard deviation.

The adjusted difference-in-differences estimates (Table 2) indicated there was no significant difference in the response between states that did and did not issue a statewide stay at-home order in encounters: total (0.29; 95%CI: −0.60, 1.18), in-person (0.01; 95%CI: −0.65, 0.66), telephone (0.26; 95%CI: −0.32, 0.84), and video (0.02; 95%CI: −0.02, 0.07). There was no differential change in the proportion of Veterans with no HUD-VASH encounter (−3.5 percentage points; 95%CI: −13.0, 6.0).

## Discussion

Veterans who lived in states that implemented stay-at-home orders did not differentially engage in HUD-VASH case management compared to Veterans who lived in states that did not implement stay-at-home orders. Rather, after the index date, case management encounters per Veteran increased relative to the prior year, regardless of whether the state instituted a stay-at-home order. The increase in encounters after the index date was primarily due to an increase in telephone encounters, and is likely credited to the effort of local HUD-VASH programs to contact Veterans. These findings demonstrate the ability of the VA, with a combination of centralized and decentralized decision making, to maintain care for a group of at-risk Veterans. VA medical centers operate under federal regulations, are able to harmonize federal and state policies, and are able to capitalize on resources to serve Veterans.

Virtual healthcare was a powerful tool to increase outreach to Veterans in HUD-VASH during the COVID-19 pandemic while limiting the risk of virus transmission. In the period after the index date, Veterans primarily engaged with HUD-VASH providers via telephone (mean number of encounters [SD] 3.41 [3.59]) and Veterans had on average only 0.07 [0.49] video encounters. However, not all encounters may be equal. In-person community and potentially video encounters can provide important clinical insight (e.g., the Veteran’s environment). Prior evaluations of HUD-VASH have shown it to be effective across heterogeneous Veterans including both genders, those with criminal histories and those with substance use disorders^14–17^. Whether this translates to virtual case management in HUD-VASH to these subpopulations deserve further study. For Veterans with certain diagnoses (e.g., dementia), a phone call or video encounter may not be as useful or stabilizing as an in-person visit. While there are no data on the effect of HUD-VASH encounter modality on housing or health outcomes, randomized trials of virtual healthcare (telephone and video) in other disease areas have found telehealth to be equivalent to in-person care^18,19^.

There is enthusiasm that after the COVID-19 pandemic subsides, telephone and video care will continue to be a powerful tool to increase HUD-VASH program reach. During the COVID-19 pandemic, the VA and HUD-VASH took several steps to ensure Veterans could continue to engage in case management. This included case managers proactively contacting Veterans during the pandemic and providing smartphones to Veterans experiencing housing insecurity^11^. However, the viability of enhanced case management outreach and providing Veterans with smartphones after the pandemic subsides is unknown. There are other challenges to sustaining telehealth in HUD-VASH. For example, a prior effort to integrate telehealth with chronic disease management in HUD-VASH was associated with enrollment, engagement and feasibility challenges^20^. In addition, Veterans’ preferences for in-person compared to telephone and video case management is unknown. Historically, Veterans in HUD-VASH or experiencing homelessness encountered barriers to engaging in virtual care which included limited access to a phone, computer, or stable internet. Cell phone ownership is common among homeless Americans, but smartphone ownership, which can facilitate video encounters, is less common^21,22^.

This study had limitations. We did not examine housing or health outcomes, there is variation in statewide stay-at-home orders, and results are specific to Veterans enrolled in HUD-VASH. In addition, we only examined utilization up to 60 days after the index date, which captures short-term effects. Finally, there may be uncontrolled confounding such as staffing of individual VA medical centers, local operations of HUD-VASH programs, states’ funding of homeless supports, and the likelihood to enact a stay-at-home order.

There was no significant difference in utilization of HUD-VASH case management between Veterans who lived in states that did and did not issue a statewide stay at-home order. Telephone and video case management in the HUD-VASH program is a viable method of care delivery that can increase program reach and access. Improving access to telephone and video-based care is a priority, but equally important is understanding which services can effectively be delivered virtually. For example, virtual care may not be the most effective modality for Veterans with housing insecurity who have challenging comorbidities. To improve telephone and video healthcare for all housing-insecure Americans, we must understand their preference for care modalities, and the effect of care modality on quality of care, housing and health outcomes.

## Supplementary information

Supplementary Information

## Data Availability

We used public data from the New York Times (https://www.nytimes.com/interactive/2020/us/coronavirus-stay-at-home-order.html. Accessed June 29, 2020), which is directly available from the New York Times or from the authors, to determine which states implemented a statewide stay-at-home (includes shelter-in-place). We linked public data with Department of Veterans Affairs administrative data (Corporate Data Warehouse and Homeless Operations Management and Evaluation System). Qualified VA investigators can contact the author for information about how to apply for access to the data used in our analyses.

## Code availability

Contact the authors for statistical code.
