# Supplementary material for: Association of statewide stay-at-home orders with utilization of case management and supportive services for veterans experiencing housing insecurity
Source: Npj Ment Health Res. 2022 Aug 26;1:9. doi: 10.1038/s44184-022-00010-x (PMC9412792; doi:10.1038/s44184-022-00010-x)

Supplementary Information: Association of Statewide Stay-at-Home Orders with Utilization of Case Management and Supportive Services for Veterans Experiencing Housing Insecurity

Supplementary Figure 1. Unadjusted average total HUD-VASH case management encounters per Veteran

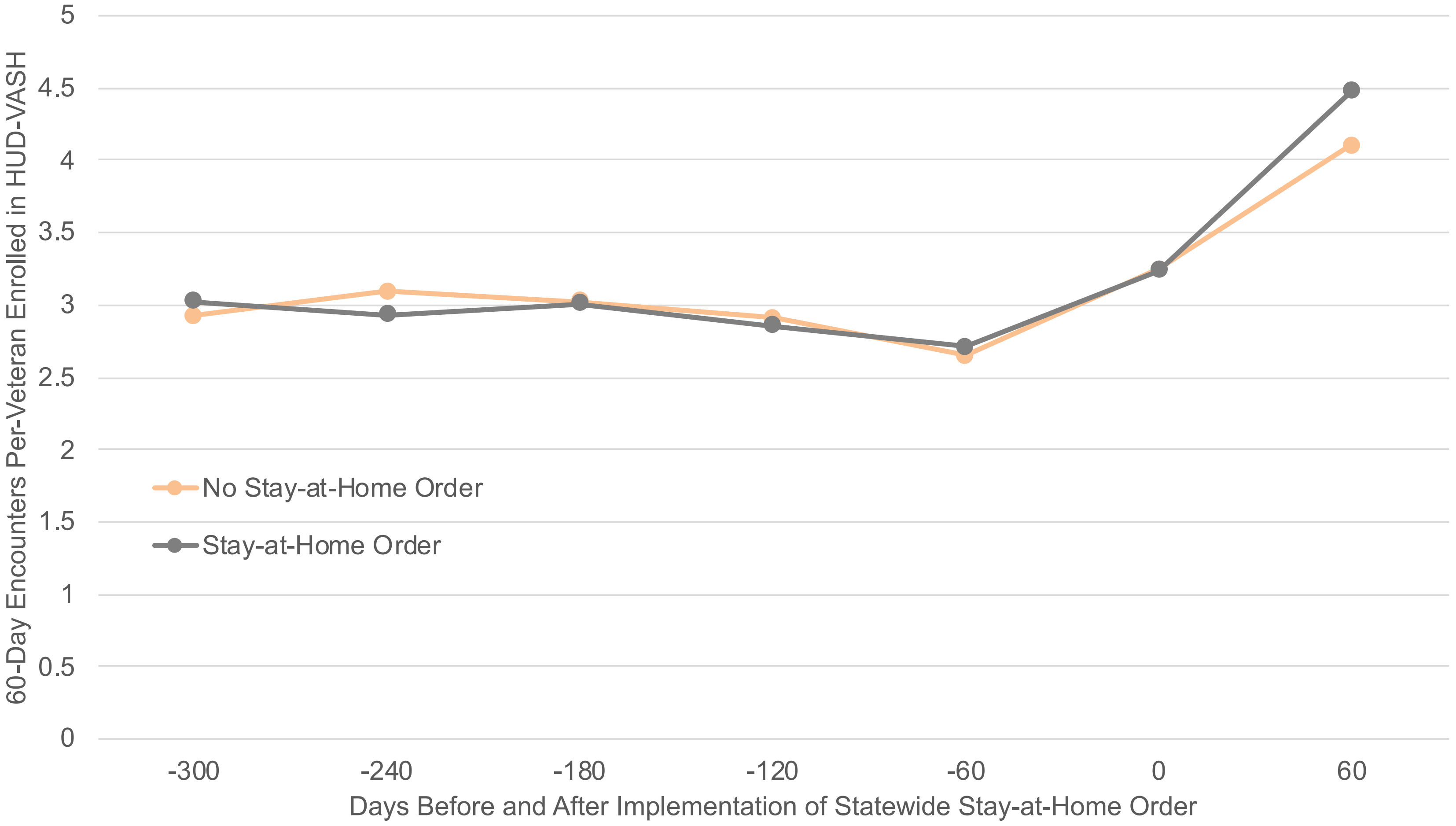

Supplementary Figure 2. Unadjusted average in-person HUD-VASH case management encounters per Veteran

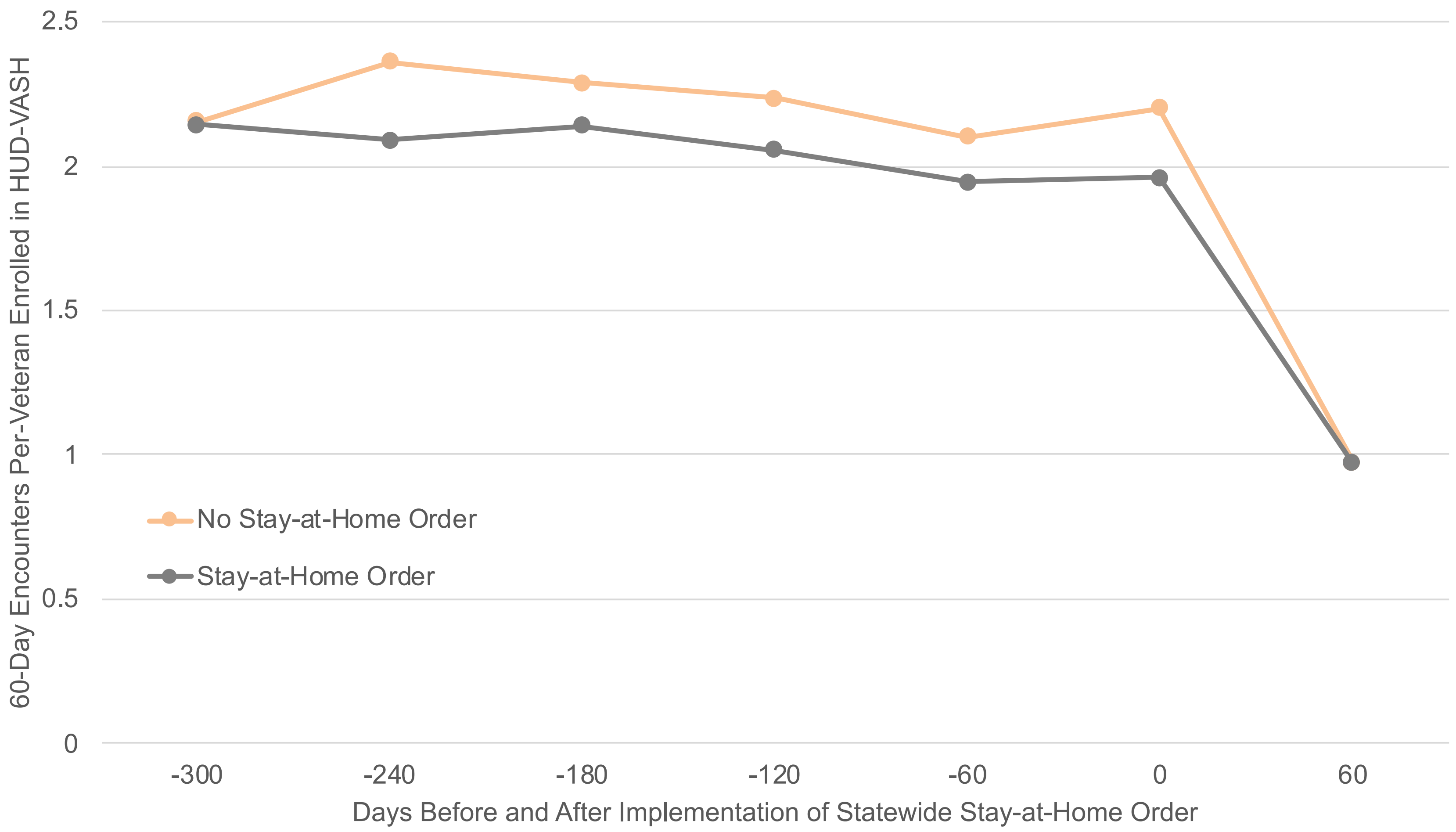

Supplementary Figure 3. Unadjusted average telephone HUD-VASH case management encounters per Veteran

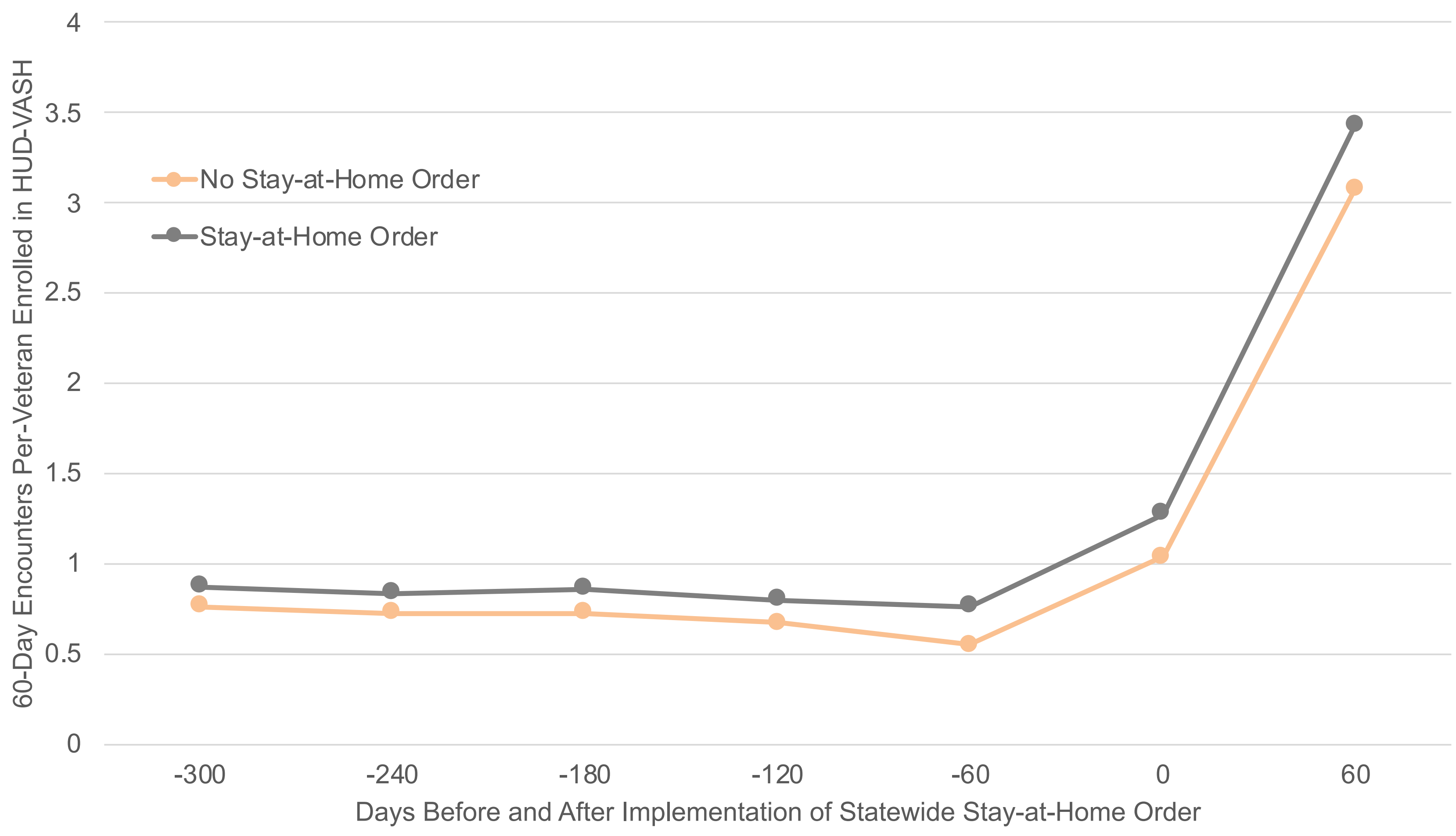

Supplementary Figure 4. Unadjusted average video HUD-VASH case management encounters per Veteran

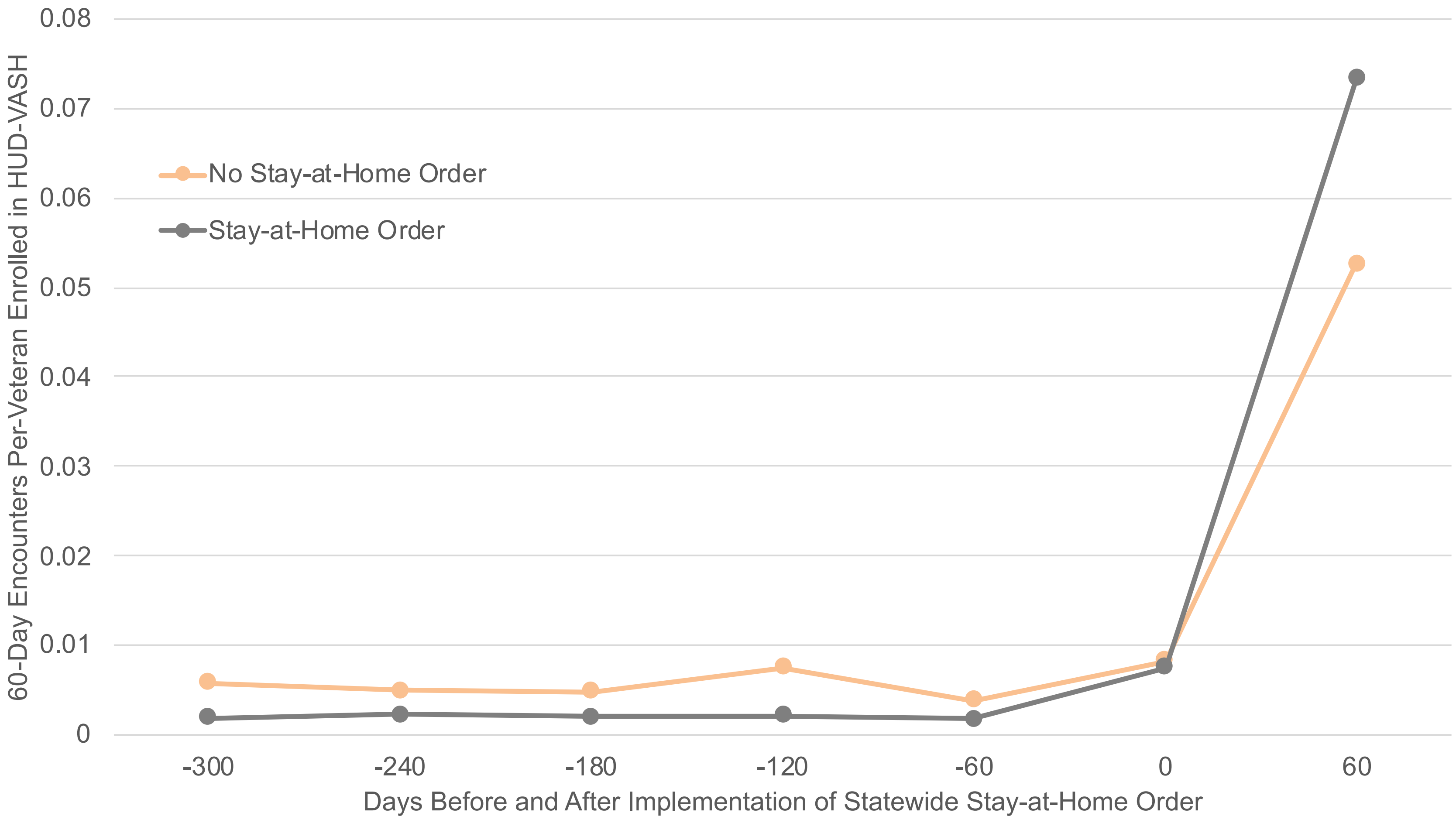

Supplementary Figure 5. Unadjusted proportion of Veterans without a HUD-VASH case management encounter

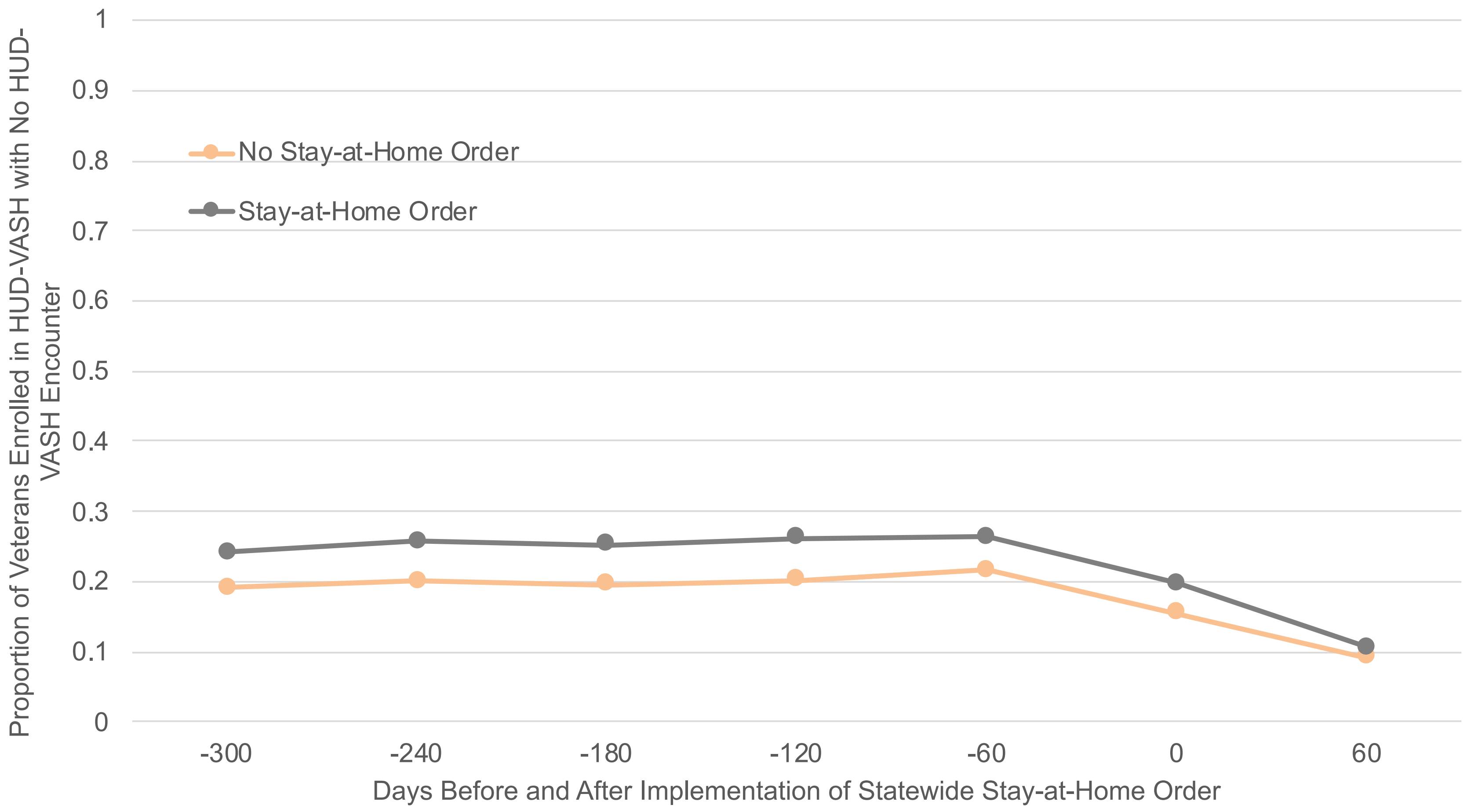

Supplement: Supplementary file 1 — Supplementary Information [file 44184_2022_10_MOESM1_ESM.pdf]
